# Does coprescribing nonsteroidal anti‐inflammatory drugs and oral anticoagulants increase the risk of major bleeding, stroke and systemic embolism?

**DOI:** 10.1111/bcp.15371

**Published:** 2022-06-08

**Authors:** Leonie S. Penner, Sean P. Gavan, Darren M. Ashcroft, Niels Peek, Rachel A. Elliott

**Affiliations:** ^1^ Manchester Centre for Health Economics, Division of Population Health, Health Services Research and Primary Care, School of Health Sciences, Faculty of Biology, Medicine and Health University of Manchester Manchester UK; ^2^ NIHR Greater Manchester Patient Safety Translational Research Centre, School of Health Sciences, Faculty of Biology, Medicine and Health University of Manchester Manchester UK; ^3^ Division of Pharmacy & Optometry, School of Health Sciences, Faculty of Biology Medicine and Health, University of Manchester Manchester UK; ^4^ Division of Informatics, Imaging and Data Science, School of Health Sciences, Faculty of Biology, Medicine and Health University of Manchester Manchester UK

**Keywords:** anticoagulants, medication safety, patient safety, pharmacoepidemiology, stroke

## Abstract

**Aims:** To examine the risk of gastrointestinal (GI) bleeding, major bleeding, stroke and systemic embolism associated with prescribing nonsteroidal anti‐inflammatory drugs (NSAIDs) to adults receiving oral anticoagulant (OAC) therapy.

**Methods:** We conducted a population‐based cohort study in adults receiving OAC therapy using linked primary care (Clinical Practice Research Datalink GOLD) and hospital (Hospital Episodes Statistics) electronic health records. We used cause‐specific Cox regression models with time‐dependent NSAID treatment in a propensity score matched population to estimate the increased risk of GI bleeding, stroke, major bleeding and systemic embolism associated with NSAID use.

**Results:** The matched cohort contained 3177 patients with OAC therapy alone and 3177 with at least 1 concomitant NSAID prescription. Compared with OAC therapy alone, concomitant prescription of NSAIDs with OACs was associated with increased risk of GI bleeding (hazard ratio [HR] 3.01, 95% confidence interval [CI] 1.63 to 5.55), stroke (HR 2.71, 95% CI 1.48 to 4.96) and major bleeding (HR 2.77, 95% CI 1.84 to 4.19). The association with systemic embolism did not reach statistical significance (HR 3.02, 95% CI 0.82 to 11.07). Sensitivity analyses indicated that the results were robust to changes in exclusion criteria and the choice of potential confounding variables.

**Conclusion:** When OACs are coprescribed with NSAIDs, the risk of adverse bleeding events increases and, simultaneously, the protective effect of OACs to prevent strokes reduces. There is a need for interventions that reduce hazardous prescribing of NSAIDs in people receiving OAC therapy.

**What is already known about this subject**

- The few studies that have investigated coprescribing nonsteroidal anti‐inflammatory drugs (NSAIDs) to patients with oral anticoagulants (OACs) indicate a possible increase in the risk of bleeding and thromboembolic events (i.e., stroke).
- Existing evidence was restricted to specific OAC types, did not investigate both event types in the same cohort and was often inconclusive.

**What this study adds**

- Coprescribing of NSAIDs increases the risk of bleeding‐related adverse drug events and reduces the effectiveness of OACs in preventing strokes.
- The substantive harm attributable to concomitant NSAIDs and OACs supports policy makers to reduce potentially hazardous prescribing in primary care

## 1 INTRODUCTION

Oral anticoagulants (OACs) and nonsteroidal anti‐inflammatory drugs (NSAIDs) are commonly associated with preventable harm and preventable drug‐related hospital admissions.^1^, ^2^, ^3^, ^4^, ^5^ A systematic review of studies reporting drugs related to preventable adverse drug events (ADEs) found NSAIDs and OACs to be involved in 11 and 8% of preventable ADEs, respectively.^1^ OACs are used in primary care for the prevention of thrombotic cardiovascular events, particularly stroke and systemic embolism. However, the mechanism that reduces the risk of thrombotic risk events also increases the risk of unwanted bleeding events. This risk of bleeding is probably increased further when NSAIDs are coprescribed with OACs.^6^, ^7^ While OACs can cause multiple types of bleeding, NSAIDs are specifically associated with gastrointestinal (GI) side effects.^8^, ^9^, ^10^, ^11^, ^12^, ^13^, ^14^, ^15^ Therefore, there is a need to better understand the impact of prescribing concomitant OACs and NSAIDs on the likelihood of subsequent bleeding events.

In recent years, NSAIDs have also been associated with an increased risk of thrombotic cardiovascular events.^16^ Given that patients treated with OACs are already at an increased risk of thrombotic cardiovascular events, evidence about the combined effect of NSAIDs and OACs on the risk of such events will be valuable to inform the management of patients in the future. Several recent studies have investigated the impact of NSAIDs on risk of stroke and systemic embolism but to date the existing evidence is inconclusive.^17^, ^18^, ^19^, ^20^

Overall, existing evidence about the impact of NSAIDs on bleeding‐related and cardiovascular ADEs is: (i) limited to specific types of OACs (warfarin alone,^19^, ^21^, ^22^, ^23^, ^24^, ^25^ 1 direct OAC [DOAC: rivaroxaban] alone^17^ or warfarin compared with 1 type of DOAC^18^, ^20^); or (ii) did not investigate a range of ADEs in the same cohort.^21^, ^22^, ^23^, ^24^, ^25^, ^26^ Therefore, the aim of this study was to examine the risk of GI bleeding, major bleeding, stroke and systemic embolism associated with prescribing NSAIDs to patients receiving OAC therapy. We quantified the increased likelihood of these ADEs in the same cohort.

## 2 METHODS

We conducted a retrospective cohort study including patients prescribed OAC therapy that compared the risk of ADEs experienced by new users of NSAIDs with people who were not prescribed NSAIDs, using electronic health records. The RECORD‐PE reporting guidelines for studies using routinely collected health data were followed^27^ (Appendix A).

### 2.1 Data resource

We used routinely collected primary care electronic health records from the Clinical Practice Research Datalink (CPRD) GOLD, linked with secondary care records from Hospital Episode Statistics Admitted Patient Care (HES APC) and mortality records from the Office for National Statistics (ONS).^28^ The CPRD GOLD collects patient‐level diagnoses and prescription data from around 600 primary care practices in the UK covering approximately 7% of the UK population.^29^, ^30^ The dataset has been shown to be broadly representative of the UK population in terms of age, sex and ethnicity,^30^, ^31^ and the validity of diagnostic coding is high.^32^, ^33^

HES APC comprises discharge details and clinical procedures for all secondary care admissions in England, and 58% of primary care practices in CPRD have agreed to data linkage with HES.^30^ Patient level data where HES linkage was available were linked to the ONS mortality data and the Index of Multiple Deprivation (IMD). The ONS mortality records provide patient‐level death records including cause of death^34^ and the IMD provides information on socioeconomic status at the postcode level of the practice.

### 2.2 Population and follow‐up

**FIGURE 1:**
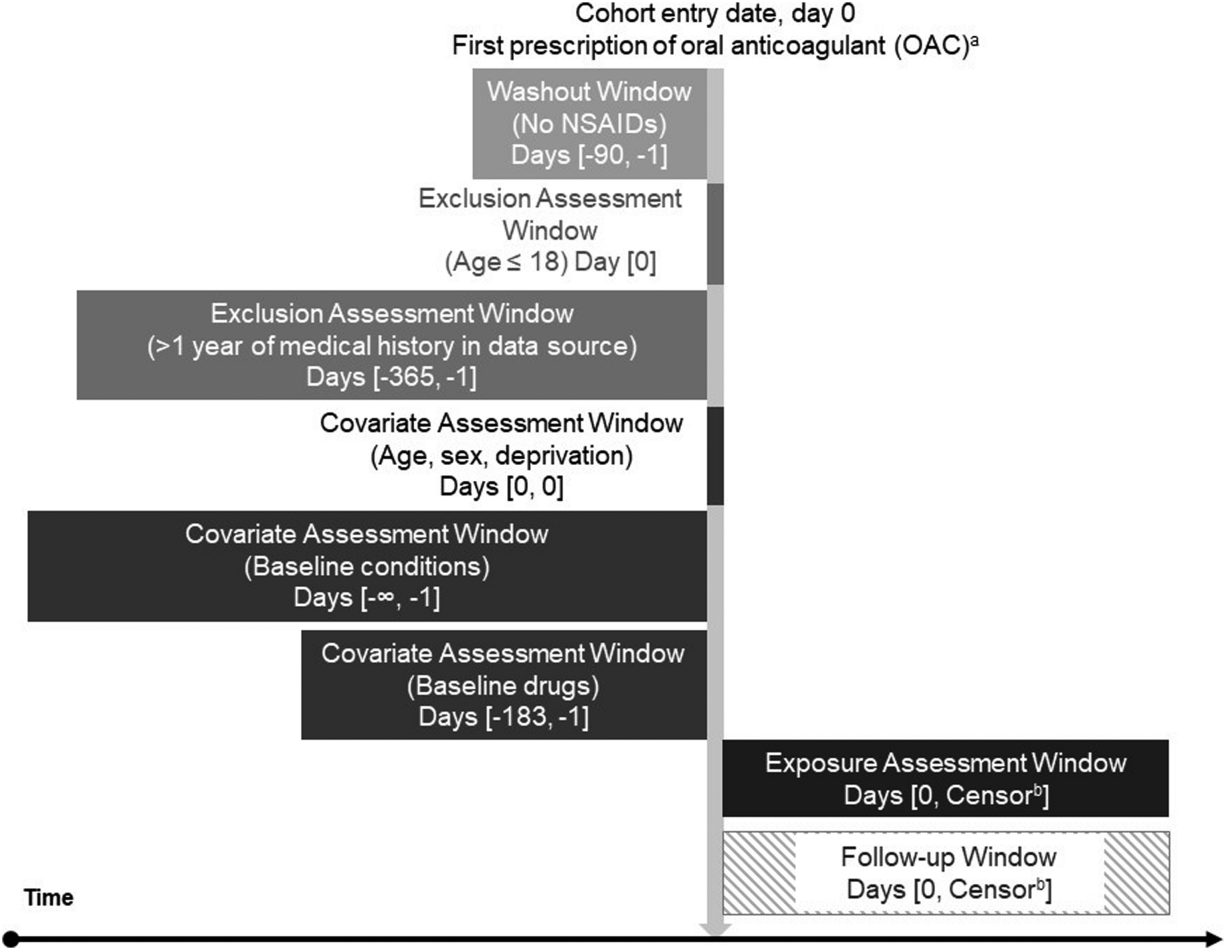
Exclusion criteria and time anchors for cohort entry and follow‐up.^a^ Treatment episodes defined by prescription day and calculated stop date. Gaps of <30 days between stop date and next prescription were bridged. Thirty days were added to the last stop date of consecutive prescriptions; ^b^ earliest of: outcome of interest, discontinuation of OAC (including 30‐d washout), death, last collection date of practice, transfer out date, end of the study period; diagram design developed and recommended as reporting standard by Schneeweiss et al.^35^

Patients were eligible for cohort entry if they had at least 1 OAC prescription (prevalent and incident users) in the study period (1 April 2007–31 December 2017), were aged ≥18 years and were registered with a CPRD‐participating up‐to‐standard practice for at least 12 months prior to cohort entry. OACs comprise: (i) vitamin‐K antagonists: warfarin, acenocoumarol and phenindione; and (ii) DOACs: rivaroxaban, edoxaban, dabigatran and apixaban. We report a visual depiction of the study design in Figure 1 and in Appendix B, as developed by Schneeweiss et al.^35^ and recommended in reporting guidelines by Patorno et al.^36^ We excluded patients if they received a prescription of an NSAID within the last 90 days before the index date (prevalent users) as proposed by Ray et al.^37^ By excluding prevalent NSAID users biases resulting from healthy user effects, time‐dependent event risks associated with NSAIDs and adjusting for intermediates can be reduced. The 90 days were considered appropriate to exclude all prevalent users of NSAIDs because 99% of prescriptions had a duration of 60 days or less plus a 30‐day grace period. In Appendix C, we describe how code lists for the drug groups were generated.

We defined start of follow‐up as the first prescription of an OAC in the study period (index date). Follow‐up ended at the date when 1 of the following occurred: (i) the OAC was stopped for >30 days; (ii) the patient left the practice; (iii) data collection for the practice ended; (iv) death; (v) the study period ended; or (vi) the patient experienced the outcome event. The prescription stop date was calculated from records on the quantity of units prescribed (e.g., tablets or capsules, and the daily dose prescribed) because it was not recorded in CPRD Gold (details in Appendix D). Treatment was assumed to be continuous if the time between stopping and starting an OAC prescription was 30 days or less. A grace period of 30 days was considered to be an appropriate time to account for overlapping repeat prescriptions.^38^, ^39^, ^40^

### 2.3 Treatment episodes and exposure

In this study, we considered patients to be exposed when they received a prescription of an NSAID. We used NSAID exposure as a time‐dependent binary variable due to the time‐varying and often short‐term nature of NSAID prescriptions. The periods where patients had a continuous NSAID prescription were defined as the exposure period (including a 30‐day grace period after the calculated prescription stop date^41^; Appendix D).

### 2.4 Outcomes

The primary outcomes were GI bleeding (including bleeding ulcers, perforated ulcers, bleeding varices, melaena, haematemesis, haemoperitoneum, haemorrhagic gastritis or unspecified GI bleeding) and stroke (including stroke, transient ischaemic attacks, intracranial haemorrhage [ICH], and cerebral infarction and strokes not specified). Secondary outcomes were major bleeding (including GI bleeding, ICH, respiratory, urinary and rectal bleeding, haemoptysis, and other unspecified bleeding) and systemic embolism (including pulmonary embolism, embolism or thrombosis of arteries). Outcome events were identified from hospital admissions (HES APC) or a death record (ONS) with a primary ICD‐10 diagnosis code related to the outcome (Appendix E).

### 2.5 Propensity score matching

Nearest neighbour propensity score matching without calliper width was used to generate balanced exposed and unexposed cohorts.^42^, ^43^ Each patient exposed to at least 1 NSAID prescription during follow‐up (labelled as NSAID user) was matched with a patient without any NSAID prescription during follow‐up (labelled as No NSAID user). The propensity score was predicted using a probit model conditioned on patient characteristics at baseline. We included baseline characteristics as potential risk factors for bleeding‐related or cardiovascular ADEs, which were identified in discussions with clinical experts: age, sex, deprivation (patient‐level IMD), ethnicity, smoking, high body mass index, alcohol dependence, severe chronic kidney disease, severe chronic liver disease, uncontrolled hypertension (>160 mmHg), bleeding event, peptic ulcer, oesophageal varices, anaemia, coronary heart disease, cerebrovascular events, peripheral artery disease, venous thromboembolism, valvular heart disease, hypertension (controlled), diabetes, chronic obstructive pulmonary disease, cancer, adverse GI events (dyspepsia, heartburn), GI inflammation, *Helicobacter pylori* infection, antiepileptic drugs (phenytoin or carbamazepine), antiplatelets, aspirin, antidepressants (selective serotonin reuptake inhibitor and tricyclic antidepressants) and corticosteroids. Data on international normalized ratio (INR) are not consistently included in CPRD records and were not included. The face validity of the identified risk factors was confirmed following discussions with 3 general practitioners. Details and derivation of code lists are described in Appendix C.

### 2.6 Statistical analysis

We estimated the effect of NSAID use on the ADEs (GI bleeding, stroke, systemic embolism, major bleeding) using a cause‐specific Cox proportional hazard model with time‐varying NSAID exposure. Results are reported as incidence rates and HRs. After the matching process created a balanced cohort and the balancing tests did not indicate otherwise (standardised difference <10%; density plot of distribution of propensity scores in the exposed and unexposed cohort), we assumed that treatment assignment (NSAID exposure) was independent of the baseline characteristics. Consequently, no further adjustment for covariates was required. The proportional hazard assumption was tested using scaled Schoenfeld residuals.^44^ All analyses were conducted using *Stata Version 16*.^45^

### 2.7 Sensitivity analysis

Sensitivity analyses explored changes in the grace period to define continuous treatment and exclusion criteria. For the first sensitivity analysis, the 30‐day grace period during continuous treatment use was extended to 60 days. The 30 days was the median NSAID prescription length. Further sensitivity analyses excluded patients with NSAID use within 30 days and 6 months prior to the index date compared with a 3‐month window in the base case analysis. In a third sensitivity analysis, an outcome washout window was applied to exclude patients if they had ever experienced the outcome before the index date.^18^

#### 2.7.1 Robustness of assumptions on conditioning on confounding variables

For the base case analysis, all variables affecting the GI bleeding outcome were considered in the propensity score model. However, including collider variables in a propensity score model can introduce a small bias.^46^ The propensity score model was therefore rerun without including the potential collider of baseline gastroprotective agent use.

Matching on the propensity score was chosen as the balancing method for the base case. In a sensitivity analysis, inverse probability treatment weighting (IPTW) on the propensity score was investigated. The IPTW method was used because it preserves the original sample size, which can be useful for studies investigating rare outcomes, such as systemic embolism. Because the probability of treatment assignment was low, resulting in a low propensity score, the use of stabilised weights has been recommended.^47^, ^48^

NSAID use was incorporated as a time‐dependent exposure in the Cox proportional hazard model. The exposure could be influenced not only by the baseline characteristics but also by changes in confounders during follow‐up. In the base case analysis, it was assumed that baseline characteristics do not change over time and no further adjustment for confounders was required in the balanced population. In the sensitivity analysis for GI events and major bleeding, time‐dependent variables were included in the proportional hazard regression model to adjust for: (i) uncontrolled hypertension (>160 mmHg); (ii) chronic kidney disease; (iii) chronic liver disease; (iv) previous bleeding event; (v) peptic ulcer; (vi) anaemia; (vii) alcoholism; (viii) age >65 years; and (ix) concomitant drugs associated with an increased bleeding risk (aspirin, antiplatelets, corticosteroids, antidepressants). For this analysis, the covariates assessed at the index date were updated before each change in the exposure status. In the respective sensitivity analysis for stroke and systemic embolism, time‐dependent variables in the CHA2DS2‐VASc stroke risk score were used: (i) congestive heart failure; (ii) uncontrolled hypertension (>160 mmHg); (iii) age >75 years; (iv) diabetes mellitus; (v) stroke/transient ischaemic attack; and (vi) vascular disease.^49^, ^50^, ^51^, ^52^

E‐values were used to estimate the minimal strength of association an unmeasured confounder must have with the outcome and the exposure to be able to explain away the observed effect.^53^ For risk ratios (RR) >1, the E‐value is calculated as follows.^53^

$$E-\text{value}=\mathrm{RR}+\sqrt{\left(\mathrm{RR}\times \left(\mathrm{RR}-1\right)\right)}.$$

To interpret the E‐value, it was compared with the magnitude of the impact of known risk factors as recommended in the published literature.^54^, ^55^

## 3 RESULTS

**FIGURE 2:**
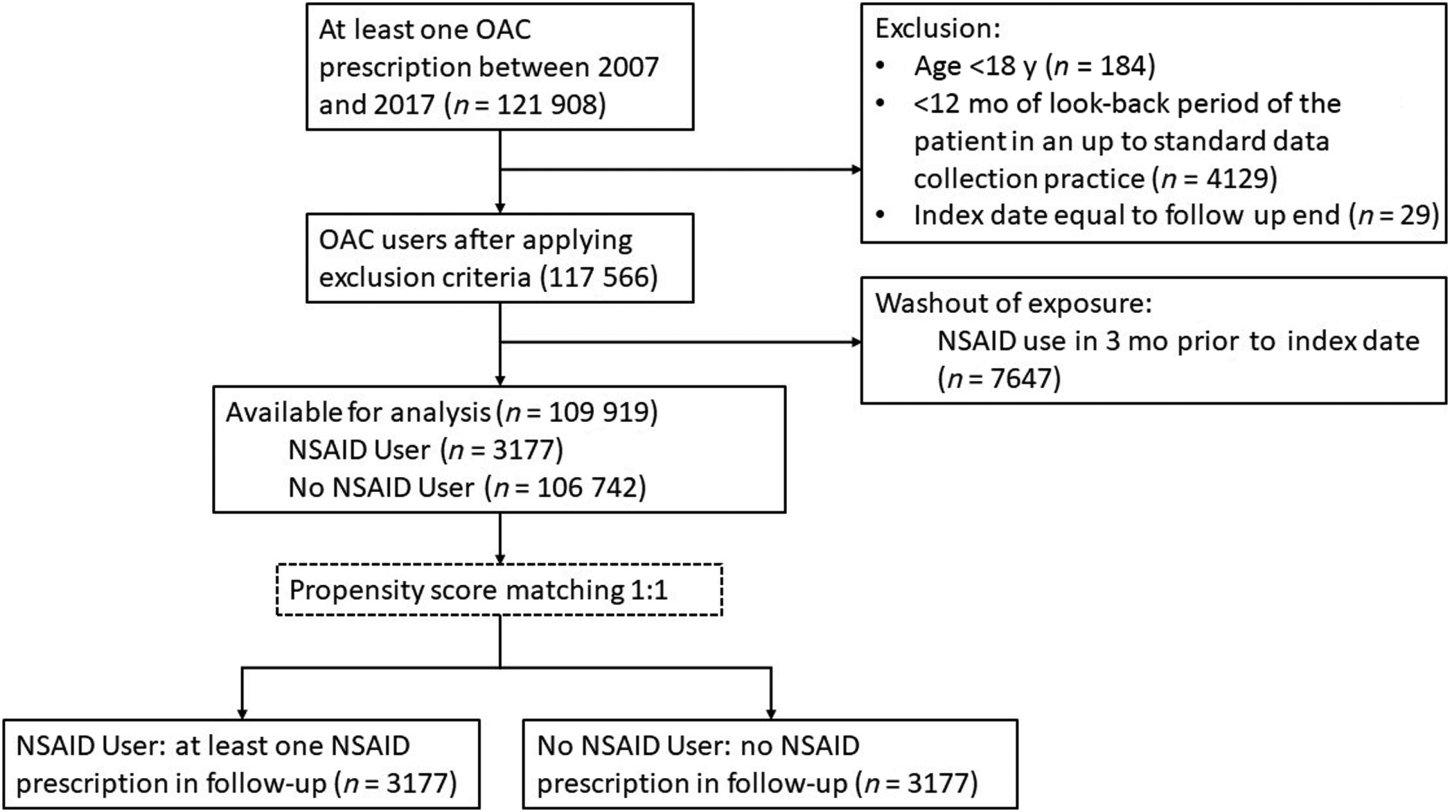
Flow chart of the patients from the dataset included in the analysis of the primary outcome. NSAID: nonsteroidal anti‐inflammatory drug; OAC: oral anticoagulant

Table 1 reports the baseline descriptive statistics for NSAID users and No NSAID users measured at the index date (i.e., first prescription of an OAC). The matched cohort consisted of 3177 NSAID users and 3177 No NSAID users. Figure 2 presents a flow diagram to illustrate how the final sample was derived. The tests conducted to test for balance between NSAID users and No NSAID users after matching indicated balanced cohorts. The mean propensity score was 0.031 (standard deviation ± 0.09) in both groups. The mean standardised difference was reduced from 3.5 to 1.8%.

**TABLE 1:** Baseline characteristics of patients in the cohort with at least 1 NSAID during follow‐up (NSAID users) and with no NSAID use (No NSAID users) before matching and after propensity score matching

Baseline characteristics	NSAID users (*n =* 3177)^a^	No NSAID users^b^	
		Before matching (*n =* 106 742)	After matching (*n =* 3177)
Propensity score, mean (SD)	0.031 (0.009)	0.029 (0.08)	0.031 (0.009)
Standardised difference, mean		3.5	1.8
Age (y), mean (SD)	70 (14)	72 (14)	70 (14)
Sex (female)	1311 (41%)	48 413 (45%)	1335 (42%)
Ethnicity			
White	2456 (77%)	82 260 (77%)	2469 (78%)
Other	15 (0%)	496 (0%)	12 (0%)
Asian	33 (1%)	940 (1%)	29 (1%)
Black	21 (1%)	711 (1%)	16 (1%)
Unknown	652 (21%)	22 335 (21%)	651 (20%)
Deprivation index (IMD)			
1 (least deprived)	671 (21%)	25 332 (24%)	697 (22%)
2	725 (23%)	24 545 (23%)	689 (22%)
3	686 (22%)	23 140 (22%)	712 (22%)
4	590 (19%)	18 647 (17%)	567 (18%)
5 (most deprived)	503 (16%)	15 014 (14%)	511 (16%)
Missing	<5 (0%)^c^	64 (0%)	<5 (0%)^c^
Smoking status			
Current smoker	505 (16%)	15 414 (14%)	525 (17%)
Ex‐smoker	1677 (53%)	54 431 (51%)	1685 (53%)
Missing	25 (1%)	937 (1%)	25 (1%)
Never smoker	970 (31%)	35 960 (34%)	942 (30%)
Blood pressure control			
Uncontrolled blood pressure	128 (4%)	4529 (4%)	116 (4%)
Controlled blood pressure	2587 (81%)	87 442 (82%)	2598 (82%)
No recorded blood pressure measurement	462 (15%)	14 771 (14%)	463 (15%)
BMI (kg/m^2^), mean (SD)	30 (6)	29 (6)	30 (7)
BMI > 30 kg/m^2^/obese	744 (23%)	19 039 (18%)	713 (22%)
*Comedication (up to 6 mo before index date)*			
Antiplatelet drug	188 (6%)	7954 (7%)	169 (5%)
Aspirin	937 (29%)	31 482 (29%)	929 (29%)
Gastroprotective agent	1147 (36%)	34 796 (33%)	1089 (34%)
Antidepressant	570 (18%)	16 280 (15%)	556 (18%)
Corticosteroids	314 (10%)	9667 (9%)	273 (9%)
Antiepileptic drug	42 (1%)	1256 (1%)	30 (1%)
*Comorbidities (ever before index date)*			
Peptic ulcer	126 (4%)	5452 (5%)	121 (4%)
Adverse GI event	772 (24%)	23 351 (22%)	726 (23%)
GI inflammation	473 (15%)	14 719 (14%)	456 (14%)
GI varices	<5 (0%)^c^	22 (0%)	<5 (0%)^c^
Alcoholism	79 (2%)	2286 (2%)	88 (3%)
Anaemia	352 (11%)	13 326 (12%)	325 (10%)
Cancer	365 (11%)	12 928 (12%)	345 (11%)
Any bleed	426 (13%)	13 505 (13%)	381 (12%)
Coronary heart disease	836 (26%)	26 726 (25%)	819 (26%)
COPD	278 (9%)	9060 (8%)	278 (9%)
Diabetes	516 (16%)	17 124 (16%)	498 (16%)
Renal disease	89 (3%)	3455 (3%)	77 (2%)
Liver disease	14 (0%)	645 (1%)	6 (0%)
Peripheral vascular disease	88 (3%)	3200 (3%)	76 (2%)
Atrial fibrillation	1660 (52%)	57 356 (54%)	1659 (52%)
Heart failure	396 (12%)	14 035 (13%)	389 (12%)
Hypertension	1674 (53%)	56 139 (53%)	1665 (52%)
Stroke	451 (14%)	17 836 (17%)	397 (12%)
Valvular heart disease	257 (8%)	11 407 (11%)	231 (7%)
Venous thromboembolism	850 (27%)	27 531 (26%)	863 (27%)

BMI: body mass index; COPD: chronic obstructive pulmonary disease; GI: gastrointestinal; IMD: Index of Multiple Deprivation; NSAID: nonsteroidal anti‐inflammatory drug; SD: standard deviation.

^a^ NSAID users: patients who received an NSAID at any time during follow‐up;

^b^ No NSAID users: patients who received no NSAID during follow‐up;

^c^ cell counts <5 are masked to avoid that patients can be identified from this analysis;

### 3.1 Increased risk of bleeding‐related and cardiovascular ADEs

The incidence rates for GI bleeding without and with NSAID use were 6.87 (95% CI 5.25 to 9.00) and 20.49 (95% CI 11.90 to 35.28) per 1000 person‐years, respectively (Table 2). The corresponding HR for GI bleeding in the presence of the NSAID was 3.01 (95% CI 1.63 to 5.55). NSAID use was also associated with an increased risk of stroke (HR 2.71 [95% CI 1.48 to 4.96]) and major bleeding (HR 2.77 [95% CI 1.84 to 4.19]). No statistically significant association with NSAID use was found for the rarer event of systemic embolism.

**TABLE 2:** Incidence rates, incidence risk ratio (IRR) and cause‐specific hazard ratio (HR) for the base case analysis on the impact of time‐dependent NSAID exposure on outcomes in patients with oral anticoagulants

Outcome	Person time		Incidence rate (95% CI)^a^		IRR (95% CI)^b^	HR (95% CI)^b^
	Exposed to NSAID^c^	Not exposed to NSAID^d^	NSAID use^c^	No NSAID use^d^		
GI bleeding	635	7710	20.49 (11.90; 35.28)	6.87 (5.25; 9.00)	2.98 (1.49; 5.54)	3.01 (1.63; 5.55)
Major bleeding	621	7532	45.06 (31.11; 65.25)	17.26 (14.53; 20.50)	2.61 (1.67; 3.95)	2.77 (1.84; 4.19)
Stroke	634	7779	20.52 (11.92; 35.33)	7.97 (6.21; 10.22)	2.57 (1.30; 4,73)	2.71 (1.48; 4.96)
Systemic embolism	636	7715	4.72 (1.52; 14.63)	1.30 (0.70; 2.41)	2.23 (0.24; 10.20)	3.02 (0.82; 11.07)

^a^ Incidence rate per 1000 person years;

^b^ unadjusted IRR and adjusted HR reported for times with concomitant NSAID and OAC use relative to times with OAC use alone;

^c^ person time exposed to NSAIDs is defined as time during which patients received both an NSAID and an OAC;

^d^ person time not exposed to NSAIDs is defined as time during which patients received an OAC and no NSAID; CI: confidence interval; NSAID: nonsteroidal anti‐inflammatory drug; OAC: oral anticoagulant.

### 3.2 Sensitivity analysis

Results of the sensitivity analysis are presented in Appendix F. Changes in the grace period to estimate continuous prescription use, the NSAID exclusion window, the exclusion of patients who had GI bleeding prior to the index date or the use of IPTW instead of propensity score matching did not result in qualitative changes in the findings (i.e., same direction of HR and no change in statistical significance). The magnitude of the estimated HR was not sensitive to the exclusion of a potential collider variable (baseline gastroprotective agent use) in the propensity score model. The sensitivity analyses described above had an equivalent impact on the estimated HRs for the major bleeding and systemic embolism outcomes (see Appendix F). For the stroke outcome, the exclusion of patients with the outcome prior to the index date and the use of IPTW did not change the direction of the effect compared with the base case but the results were not significant anymore.

### 3.3 E‐values

For GI bleeding, an E‐value of 5.45 (lower bound: 2.64) was calculated. The calculated E‐values, and the impact of other variables on the risk of the outcome conditional on NSAID exposure, are reported in Appendix G1, G2. The observed risk ratio could be explained away by an unmeasured confounder that was associated with both the NSAID prescription and GI bleeding by a risk ratio of at least 5.45‐fold each.^53^ In comparison, the maximum impact that a measured confounder had on the outcome conditional on NSAID exposure was 2.80, for the HR for peptic ulcer (Appendix G1, G2). Hence, for major bleeding and stroke the E‐value was 4.84 (lower bound, 3.08) and 4.84 (lower bound, 2.32). The respective risk factors had a lower estimated association with the outcome conditional on NSAID use compared with the mean E‐value. The HRs for 1 of the risk factors for GI bleeding (peptic ulcer HR: 2.80) and stroke (previous stroke: 3.80) had an association with the outcome conditional on NSAID use that was larger than the lower bound of the E‐value. For systemic embolism no E‐value was calculated because the HR was nonsignificant.

## 4 DISCUSSION

Prescribing NSAIDs to patients with OAC therapy was associated with an increased incidence of GI bleeding, major bleeding and stroke compared with anticoagulated patients without concomitant NSAID treatment. We did not find a significant association between NSAID use and an increased risk of systemic embolism. Our study provides valuable evidence to justify the need to address potentially hazardous prescribing in primary care in order to improve patient health outcomes.

Three studies have investigated the impact of NSAIDs on both the risk of bleeding‐related and cardiovascular ADEs previously.^18^, ^19^, ^20^ Dalgaard et al.^20^ and Kent et al.^18^ utilised data from randomized controlled trials comparing a DOAC with warfarin, and Lamberts et al.^19^ used Danish registry data. In contrast to our study, which includes any OAC, all patient cohorts in these previous studies were restricted to specific OAC types. The data analysis by Kent et al., restricted to dabigatran and warfarin users, identified an increase in GI bleeding risk (HR 1.81, 95% CI 1.35 to 2.43) in the subgroup of reported NSAID and non NSAID users,^18^ Dalgaard et al., restricted to apixaban and warfarin users, did not identify such an increase (HR 1.08, 95% CI 0.64 to 1.82).^20^ Lamberts et al., restricted to warfarin and phenprocoumon users, reported an adjusted HR of 3.54 (95% CI 3.29 to 3.82) for GI bleeding. Stroke events in the data used by Kent et al. were also significantly increased with NSAID use (HR 1.55, 95% CI 1.11 to 2.16) as was systemic embolism (HR 2.43, 95% CI 1.08 to 5.46). Dalgaard et al. and Lamberts et al. investigated the combined risk increase associated with NSAIDs of stroke/systemic embolism but did not report significant results. Differences among these study results could be due to different OAC types or the definitions of how NSAID exposure was reported and defined. In the studies by Kent et al. and Dalgaard et al., NSAID use was based on self‐report during regular meetings, lacking a clear definition of start and stop dates of NSAID treatment as was applied in our study. The results from this study build on the earlier findings of Lambert et al. by balancing patient characteristics at baseline, which may mitigate the risk of overfitting a regression model with mediator covariates, such as hypertension, chronic renal failure, liver failure, previous stroke, history of alcohol misuse, previous bleeding event, heart failure, diabetes, previous embolism or vascular disease.

### 4.1 Strengths and limitations

This is the first study examining the increased risk of bleeding‐related and cardiovascular ADEs associated with NSAIDs in the same population with any type of OAC. It was also the first UK study to estimate the impact of NSAIDs on ADEs in patients with concomitant OAC therapy. A major strength of our study was the availability of nationally representative linked primary and secondary care data with detailed information on patient demographics and potential confounding variables, such as life‐style factors, comorbidities and prescriptions. Earlier studies were mostly based on administrative claims databases^19^, ^23^, ^56^, ^57^, ^58^ or data from 1 hospital alone with no primary care records available.^24^, ^59^, ^60^ In contrast to earlier studies,^19^, ^21^, ^22^, ^23^, ^25^, ^60^, ^61^ the cohort in this study was also not restricted to warfarin users only or to a single indication for OAC therapy, such as atrial fibrillation.^18^, ^20^, ^62^ Both can contribute to a reduced generalisability of results because warfarin only represented 26% of all OAC prescriptions in England in 2019,^63^ and approximately only 50% of OACs are prescribed for atrial fibrillation in England.^40^ As a consequence of the observational study design, 1 of the limitations of this study is the risk of unmeasured confounding due to variables not recorded in the dataset. In this study, the risk of confounding was mitigated by design, analysis and quantified to some extent using E‐values.^53^ Known risk factors were less associated with the outcome than the mean E‐value. For major bleeding, this was also true for the lower bound of the E‐value. The association of the strongest predictors of stroke and GI bleeding identified within the dataset were lower than the identified mean E‐value but not the lower bound of that E‐value. However, key risk factors, such as stroke or peptic ulcer respectively, were measured in the CPRD and were accounted for in the analysis. However, there may be potential confounders that were not measured in the CPRD. For example, poor INR control is a potential confounder for bleeding events but this is not routinely collected in the CPRD.^38^, ^64^ In the literature, poor INR control compared with excellent INR control has been associated with a HR of 1.99 (95% CI 1.79 to 2.25) for major bleeding events which is considerably smaller than the E‐value we estimated for GI bleeding or major bleeding.^65^ A multinational case–control study found the diet risk score (odds ratio [OR] 1.35), regular physical activity (OR 0.69), psychosocial stress (OR 1.30) and the ratio of apolipoproteins B to A1 (OR 1.89), which are all not available in the linked dataset, not to be associated with stroke by a risk ratio greater than the lower bound of the E‐value.^66^ The risk of unmeasured confounding in this study was therefore considered to be low. A second limitation was the potential for residual confounding due to measurement error. In routinely collected data, measurement errors, such as misclassified diagnoses or misclassified ICD‐codes used to screen for outcomes, cannot be ruled out. To minimise the uncertainty around the outcome events, the code lists used to identify the events were based on extensive literature searches by the lead author and a second researcher. The code lists went through a rigorous consensus process with GPs and pharmacists to identify a comprehensive set of codes for the relevant outcomes. The ICD‐codes identified from the reviews were combined and the full set was sent to 3 GPs for initial comments and identification of relevant codes for inclusion. This review was followed by multiple telephone conferences with the 3 GPs and 2 pharmacists to discuss codes with inconsistent initial feedback until consensus was achieved. A third limitation was the potential for unmeasured NSAID exposure. NSAIDs are often sold over the counter (OTC) in pharmacies. These OTC prescriptions were not recorded in the CPRD. This could have resulted in the inclusion of new NSAID users that were already on OTC NSAIDs and bias the exposure definition. Patients who are not newly prescribed NSAIDs in the cohort could introduce healthy user bias that could have underestimated the true harm from NSAIDs. If patients who were not exposed to NSAIDs according to their CPRD records acquired OTC NSAIDs, the measured effect of NSAID exposure on harm outcomes would have been underestimated. In the CPRD, the indication for NSAID use was also not recorded. While the indication of NSAID use might have had an impact on the duration of treatment use, it can only confound the results if it is also related to an increased bleeding risk. In the propensity score matched cohort, we balanced out measurable baseline characteristics that could have been related to the NSAID prescription and bleeding events. Consequently, we assumed no further need to account for indications of NSAID use.

### 4.2 Suggestions for research

While this study contributes to the sparse literature around harm from hazardous prescribing, there are many other hazardous prescribing events.^67^ The Department of Health and Social Care published a list of prescribing safety indicators of high relevance that describe situations such as the combination of OACs and NSAIDs.^68^ The same methods used in this study can be applied to explore the health consequences of other prescribing safety indicators. Future research could also aim to extrapolate long‐term health outcomes, such as quality of life, or health care resource use associated with hazardous prescribing.

## 5 CONCLUSION

Using a large cohort of linked primary and secondary care electronic health records, we found that when NSAIDs are prescribed to patients with OAC therapy, the risk of adverse bleeding events increases and, simultaneously, the protective effect of OACs to prevent strokes reduces. Evidence of harm from hazardous prescribing reinforces the need to act to reduce NSAID use in patients receiving OAC therapy. Decision‐makers and clinicians should support interventions designed to reduce hazardous prescribing to improve patient health outcomes.

## COMPETING INTERESTS

D.M.A. reports research funding from Abbvie, Almirall, Celgene, Eli Lilly, Janssen, Novartis, UCB and the Leo Foundation outside the submitted work. N.P. reports research funding from Novo Nordisk outside the submitted work. R.A.E. reports research funding from Takeda, Janssen and CSL Behring outside the submitted work. There are no financial relationships with any organisations that might have an interest in the submitted work. No other relationships or activities that could appear to have influenced the submitted work.

## ETHICS APPROVAL

Access to the linked dataset was approved by the Independent Scientific Advisory Committee for MHRA database research (ISAC). The protocol (No 18_235) was approved on 12 August 2018.

## CONTRIBUTORS

L.P. conducted this study as part of her PhD thesis. All authors were involved in the study design. L.P. conducted the data analysis and wrote the initial draft. All authors contributed to further drafts and approved the final manuscript.

## Data Availability

The clinical codes used in this study are published on Clinicalcodes.org. Electronic health records are, by definition, considered *sensitive* data in the UK by the Data Protection Act and cannot be shared via public deposition because of information governance restriction in place to protect patient confidentiality. Access to data is available only once approval has been obtained through the individual constituent entities controlling access to the data. The data can be requested via application to the Clinical Practice Research Datalink (www.cprd.com). Requests to access these datasets should be directed to enquiries@cprd.com.

## Appendix A Checklists for reporting in observational cohort studies

**TABLE A1:** The RECORD, STROBE and RECORD‐PE statements and sections the items are referred to

Item no	STROBE items	RECORD items	RECORD‐PE items	Section
*Title and abstract*				
1	(a) Indicate the study's design with a commonly used term in the title or the abstract. (b) Provide in the abstract an informative and balanced summary of what was done and what was found.	1.1: The type of data used should be specified in the title or abstract. When possible, the name of the databases used should be included. 1.2: If applicable, the geographical region and timeframe within which the study took place should be reported in the title or abstract. 1.3: If linkage between databases was conducted for the study, this should be clearly stated in the title or abstract.		Abstract
*Introduction*				
Background rationale				
2	Explain the scientific background and rationale for the investigation being			Introduction
Objectives				
3	State specific objectives, including any prespecified hypotheses.			Introduction
*Methods*				
Study design				
4	Present key elements of study design early in the paper		4.a: Include details of the specific study design (and its features) and report the use of multiple designs if used. 4.b: The use of a diagram(s) is recommended to illustrate key aspects of the study design(s), including exposure, washout, lag and observation periods, and covariate definitions as relevant	Methods, Appendix B
Setting				
5	Describe the setting, locations and relevant dates, including periods of recruitment, exposure, follow‐up and data collection.			Methods, Appendix B
Participants				
6	(a) Cohort study—Give the eligibility criteria, and the sources and methods of selection of participants. Describe methods of follow‐up. Case–control study—Give the eligibility criteria, and the sources and methods of case ascertainment and control selection. Give the rationale for the choice of cases and controls. Cross sectional study—Give the eligibility criteria, and the sources and methods of selection of participants. (b) Cohort study—For matched studies, give matching criteria and number of exposed and unexposed. Case–control study—For matched studies, give matching criteria and the number of controls per case.	6.1: The methods of study population selection (such as codes or algorithms used to identify participants) should be listed in detail. If this is not possible, an explanation should be provided. 6.2: Any validation studies of the codes or algorithms used to select the population should be referenced. If validation was conducted for this study and not published elsewhere, detailed methods and results should be provided. 6.3: If the study involved linkage of databases, consider use of a flow diagram or other graphical display to demonstrate the data linkage process, including the number of individuals with linked data at each stage.	6.1.a: Describe the study entry criteria and the order in which these criteria were applied to identify the study population. Specify whether only users with a specific indication were included and whether patients were allowed to enter the study population once or if multiple entries were permitted. See explanatory document for guidance related to matched designs.	Methods, Appendix B
Variables				
7	Clearly define all outcomes, exposures, predictors, potential confounders and effect modifiers. Give diagnostic criteria if applicable.	7.1: A complete list of codes and algorithms used to classify exposures, outcomes, confounders and effect modifiers should be provided. If these cannot be reported, an explanation should be provided.	7.1.a: Describe how the drug exposure definition was developed. 7.1.b: Specify the data sources from which drug exposure information for individuals was obtained. 7.1.c: Describe the time window(s) during which an individual is considered exposed to the drug(s). The rationale for selecting a particular time window should be provided. The extent of potential left truncation or left censoring should be specified. 7.1.d: Justify how events are attributed to current, prior, ever or cumulative drug exposure. 7.1.e: When examining drug dose and risk attribution, describe how current, historical or time on therapy are considered. 7.1.f: Use of any comparator groups should be outlined and justified. 7.1.g: Outline the approach used to handle individuals with >1 relevant drug exposure during the study period.	Methods
Data sources/measurement				
8	For each variable of interest, give sources of data and details of methods of assessment (measurement). Describe comparability of assessment methods if there is >1 group.		8.a: Describe the healthcare system and mechanisms for generating the drug exposure records. Specify the care setting in which the drug(s) of interest was prescribed.	Appendix B
Bias				
9	Describe any efforts to address potential sources of bias.			Methods
Study size				
10	Explain how the study size was arrived at.			Figure 2
Quantitative variables				
11	Explain how quantitative variables were handled in the analyses. If applicable, describe which groupings were chosen, and why.			Appendix B
Statistical methods				
12	(a) Describe all statistical methods, including those used to control for confounding. (b) Describe any methods used to examine subgroups and interactions. (c) Explain how missing data were addressed. (d) Cohort study—If applicable, explain how loss to follow‐up was addressed. Case–control study—If applicable, explain how matching of cases and controls was addressed. Cross sectional study—If applicable, describe analytical methods taking account of sampling strategy. (e) Describe any sensitivity analyses.		12.1.a: Describe the methods used to evaluate whether the assumptions have been met. 12.1.b: Describe and justify the use of multiple designs, design features or analytical approaches.	Methods
Data access and cleaning methods				
12		12.1: Authors should describe the extent to which the investigators had access to the database population used to create the study population. 12.2: Authors should provide information on the data cleaning methods used in the study.		Methods, Appendix D
Linkage				
12		12.3: State whether the study included person level, institutional level, or other data linkage across 2 or more databases. The methods of linkage and methods of linkage quality evaluation should be provided.		Methods
*Results*				
Participants				
13	(a) Report the numbers of individuals at each stage of the study (e.g., numbers potentially eligible, examined for eligibility, confirmed eligible, included in the study, completing follow‐up and analysed). (b) Give reasons for nonparticipation at each stage. (c) Consider use of a flow diagram.	13.1: Describe in detail the selection of the individuals included in the study (i.e., study population selection) including filtering based on data quality, data availability and linkage. The selection of included individuals can be described in the text or by means of the study flow diagram.		Methods, Figure 2
Descriptive data				
14	(a) Give characteristics of study participants (e.g., demographic, clinical, social) and information on exposures and potential confounders. (b) Indicate the number of participants with missing data for each variable of interest. (c) Cohort study—Summarise follow‐up time (e.g., average and total amount).			Results, Table 1
Outcome data				
15	Cohort study—Report numbers of outcome events or summary measures over time. Case–control study—Report numbers in each exposure category, or summary measures of exposure. Cross sectional study—Report numbers of outcome events or summary measures.			Results
Main results				
16	(a) Give unadjusted estimates and if applicable, confounder adjusted estimates and their precision (e.g., 95% confidence intervals). Make clear which confounders were adjusted for and why they were included. (b) Report category boundaries when continuous variables are categorised. (c) if relevant, consider translating estimates of relative risk into absolute risk for a meaningful time period.			Results, methods
Other analyses				
17	Report other analyses done—e.g., analyses of subgroups and interactions and sensitivity analyses.			Results, Appendix F
*Discussion*				
Key results				
18	Summarise key results with reference to study objectives.			Discussion
Limitations				
19	Discuss limitations of the study, considering sources of potential bias or imprecision. Discuss both direction and magnitude of any potential bias.	19.1: Discuss the implications of using data that were not created or collected to answer the specific research question(s). Include discussion of misclassification bias, unmeasured confounding, missing data and changing eligibility over time, as they pertain to the study being reported.	19.1.a: Describe the degree to which the chosen database(s) adequately captures the drug exposure(s) of interest.	Discussion
Interpretation				
20	Give a cautious overall interpretation of results considering objectives, limitations, multiplicity of analyses, results from similar studies and other relevant evidence.	20.a: Discuss the potential for confounding by indication, contraindication or disease severity or selection bias (healthy adherer/sick stopper) as alternative explanations for the study findings when relevant.		Discussion
Generalisability				
21	Discuss the generalisability (external validity) of the study results.			Discussion
Other information				
Funding				
22	Give the source of funding and the role of the funders for the present study and if applicable, for the original study on which the present article is based.			Acknowledgments
Accessibility of protocol, raw data and programming code				
23		22.1: Authors should provide information on how to access any supplemental information such as the study protocol, raw data or programming code.		Acknowledgments

RECORD: reporting of studies conducted using observational routinely collected data; RECORD‐PE = RECORD for pharmacoepidemiological research; STROBE: strengthening the reporting of observational studies in epidemiology. This checklist has been duplicated from Table 1 in BMJ 2018;363:k3532, as a standalone document for readers to print out or fill in electronically.

## Appendix B Details on the study design and sources for covariates

**TABLE B1:** Temporal anchors for the observational cohort study

Term	Definition
Study period	1 April 2007–31 December 2017
Cohort entry	First OAC prescription in study period in patients ≥18 y, at least 12 mo of follow‐up in an up to standard practice; cohort entry = index date
Outcome event date	Day of first GI bleeding, major bleeding, stroke or systemic embolism as recorded in hospital records (episode start date in HES inpatient data)
Washout window for denominator	Not applicable, because the cohort included all OAC users
Washout window for exposure	NSAID prescriptions prescribed 90 d before index date; different assumptions tested in sensitivity analysis
Washout window for outcome	Not applicable; different assumptions tested in sensitivity analysis
Exclusion assessment window	At baseline, index date
Covariate assessment window	For comorbidities, all entries before index date were considered. For concomitant drugs, prescriptions 6 mo before index date were assessed; BMI and blood pressure were identified from records 12 mo before index date
Exposure assessment window	Time varying exposure of NSAID use during continuous OAC use
Follow‐up start	Start of follow‐up at index date (first OAC prescription in study period)
Follow‐up end	First occurrence of either: (i) outcome event; (ii) transfer out date; (iii) last collection data in CPRD GOLD; (iv) death date (ONS); calculated OAC stop date with a 30‐d grace period of last consecutive OAC prescription; or (v) end of study period.

BMI: Body mass index; CPRD GOLD: Clinical practice research datalink GOLD; GI: gastrointestinal; HES: hospital episodes statistics; NSAID: nonsteroidal anti‐inflammatory drugs; OAC: Oral anticoagulant; ONS: Office for National Statistics.

## Appendix C Covariate definitions and sources of code lists

**TABLE C1:** List of potential covariates with details on where code lists were derived from and where the records were extracted from

Covariate	Definition	Source of code list	Source of data
*Patient specific information*			
Sex	Gender (male or female)	N/A	From CPRD records at baseline
Deprivation	Index of Multiple Deprivation (IMD). Level 1 (least deprived) to level 5 (most deprived)	N/A	From linked patient level IMD records
Ethnicity	White, Asian, black, other and unknown		From HES and CPRD records
Age	Age in the year of index date		From year of birth record in CPRD
Smoking	Smoking status was subdivided into current smoker, ex‐smoker, never smoker and missing smoking status.	Code lists linking records with smoking status were derived from colleagues and entity types 4 and 23 were used.	From additional, clinical and referral file records in CPRD.
Alcohol dependence	Alcohol dependence includes heavy drinkers and excludes moderate alcohol consumption.	Code lists were previously used by Vinogradova et al.^40^ and provided by the authors.	From clinical CPRD records.
High BMI	BMI > 30 kg/m^2^ (obese)	Entity types 13 and 14 were used	From additional and clinical records in CPRD.
*Comorbidities*			
Renal insufficiency	Chronic kidney disease stage 4 or worse and renal insufficiencies with similar severity, chronic dialysis or transplant	Code lists were previously used^40^ and provided by the authors.	From clinical CPRD records.
Liver disease	Severe liver disease (code list was reviewed by GPs and found to affect bleeding risk)	Code lists were previously used^40^ and provided by the authors.	From clinical CPRD records.
Atrial fibrillation (AF)	Diagnosis of AF	Code lists were previously used^40^ and provided by the authors.	From clinical CPRD records.
Coronary heart disease (CHD)	Diagnosis of CHD, heart failure (HF), myocardial infarction or angina	Code lists were previously used^40^ and provided by the authors. Codes for HF were used as identified by PRIMIS.	From clinical CPRD records.
Cerebrovascular disease	Diagnosis of stroke and transient ischaemic attack	Codes were used as identified by PRIMIS.	From clinical CPRD records.
Peripheral artery disease (PAD)	Diagnosis of PAD	Code list was previously published^69^	From clinical CPRD records.
Venous thrombo‐embolism (VTE)	Diagnosis of pulmonary embolism or deep vein thrombosis	Codes were used as identified by PRIMIS	From clinical CPRD records.
Valvular heart disease	Diagnosis of valvular heart disease	Code lists were previously used^40^ and provided by the authors.	From clinical CPRD records
Hypertension	Diagnosis of hypertension	Code lists were previously used^40^ and provided by the authors.	From clinical CPRD records.
Uncontrolled hypertension	Blood pressure measurements >160 mmHg	Entity type 1 was used [the closest record within 12 mo before the index date]	From additional and clinical records in CPRD.
Labile International Normalized Ratio		N/A not reported consistently	N/A
Diabetes	Diagnosis of diabetes (type 1 and type 1)	Code lists were previously used^40^ and provided by the authors.	From clinical CPRD records.
Chronic obstructive pulmonary disorder (COPD)	Diagnosis of COPD	Codes were used as identified by PRIMIS.	From clinical CPRD records.
Cancer	Diagnosis of the 12 most common cancer types (includes most GI cancers)	Code lists were previously used^40^ and provided by the authors.	From clinical CPRD records
Bleeding event	Diagnosis of bleeding events were considered too minor to be included when recorded in CPRD but were included when identified as primary diagnosis in hospital records	Codes were used as identified by PRIMIS and combined with codes previously used^40^ and provided by the authors. From HES records the outcome codes for major bleeding events were used.	From clinical CPRD records.
Peptic ulcer	Diagnosis of peptic ulcer disease (excluded were perforated and haemorrhagic ulcers, which were included as bleeding events)	Codes were used as identified by PRIMIS.	From clinical CPRD records.
Adverse GI event	Diagnosis of dyspepsia and heartburn	Code lists were previously used^40^ and provided by the authors.	From clinical CPRD records.
GI inflammation	Diagnosis of gastritis, duodenitis and oesophagitis	Code list was developed using the code browser and PCD search (keywords: *gastrit*, *duodeni*, *oesophagi*)	From clinical CPRD records.
*Helicobacter pylori*		Code list was developed using the code browser and PCD search (keywords: *pylori* *helicob*)	From clinical CPRD records.
Oesophageal varices	Diagnosis of oesophageal varices	Code lists were previously used in^40^ and provided by the authors.	From clinical CPRD records.
Anaemia	Diagnosis of anaemia	Code list was developed using the code browser and PCD search (keywords: anemia, anaemia, *anaem*)	From clinical CPRD records.
*Medications*			
NSAID	Prescriptions of systemic NSAIDs (excluding aspirin)	Code list was developed using the code browser and PCD search (keywords: All drug names of this group found in BNF)	From therapy CPRD records.
Aspirin	Prescriptions of systemic aspirin	Code list was developed using the code browser and PCD search (keywords: All drug names of this group found in BNF)	From therapy CPRD records.
Antiplatelet	Prescriptions of systemic antiplatelets	Code list was developed using the code browser and PCD search (keywords: All drug names of this group found in BNF)	From therapy CPRD records.
OAC	Prescriptions of systemic warfarin, phenprocoumon, phenindione and DOACs	Code list was developed using the code browser and PCD search (keywords: All drug names of this group found in BNF)	From therapy CPRD records.
Anti‐depressants	Prescriptions of systemic SSRI and TCAs	Code lists were previously used^40^ and provided by the authors.	From therapy CPRD records.
Corticosteroid	Prescriptions of systemic corticosteroids (excluding inhaled corticosteroids)	Code lists were previously used^40^ and provided by the authors.	From therapy CPRD records.
Anti‐convulsants	Prescriptions for phenytoin or carbamazepine	Code lists were previously used^40^ and provided by the authors.	From therapy CPRD records.
GPA	Prescriptions for systemic formulations of H2‐receptor antagonists, PPIs and misoprostol	Code list was developed using the code browser and PCD search (keywords: All drug names of this group found in BNF)	From therapy CPRD records.

BMI: body mass index; BNF: British National Formulary; CHA2DS2‐VASc: stroke risk score for patients with atrial fibrillation; CPRD: Clinical Practice Research Datalink; DOAC: direct oral anticoagulant; GI: gastrointestinal; GPA: gastroprotective agent; HES: Hospital Episodes Statistics; NSAID: nonsteroidal anti‐inflammatory drug; PCD search: CPRD code generation programme in Stata by Kontopantelis (2015)^68^; PPI: proton‐pump inhibitor; PRIMIS: organisation based at University of Nottingham https://www.nottingham.ac.uk/primis/index.aspx; SSRI: selective serotonin reuptake inhibitor; TCA: tricyclic antidepressant.

## Appendix D

Identifying periods of continuous medication use

Definitions for follow‐up as well as for exposure were based on the assessment of continuous medication use. Continuous medication use was described as consecutive prescriptions of the same drug class with a grace period of 30 days between calculated stop of the prescription and the start date of the next. Because the stop date was not recorded in Clinical Practice Research Datalink; (CPRD), it was calculated from available records on quantity of units, e.g., tablets or capsules, and the daily dose prescribed. Pye et al. criticized the lack of guidance on how to process medication data and the authors developed an algorithm guiding how to report the processing of prescription data in CPRD to increase transparency.^70^ The first step to report was how missing and implausible records were cleaned. For direct oral anticoagulants (DOACs) and nonsteroidal anti‐inflammatory drugs (NSAIDs) recorded daily doses were considered implausible if dose units were not in tablets but in mg or mL because these generated extreme durations and could not be used in the calculation of the prescription length. Daily doses of zero were also assumed to be implausible. All implausible daily doses were set to missing. Those daily doses missing or set to missing were replaced by the standard dose described in the British National Formulary (BNF) if this did not vary for different indications or formulations.^71^, ^72^ For DOACs standard doses were identified from the BNF and replaced the missing daily doses. NSAIDs with many possible daily doses for the same strength were kept as missing.

Then, stop dates of each prescription were generated by adding the prescription length to the prescription date. The prescription length was calculated by dividing the prescribed quantity by the daily dose. If daily doses were recorded in the CPRD for >50% of the prescriptions of a drug group, the prescription length was calculated using the recorded daily dose and the recorded quantity. This was the case for NSAIDs with a missingness of all NSAID substances of 18% and for DOACs with missingness ranging from 22 to 27% depending on the substance.

The use of warfarin or other vitamin K antagonists (VKAs) was different. In practice, daily doses are adapted according to International Normalized Ratio measurements.^72^ Patients can have prescriptions of warfarin with different strength tablets prescribed together, so that the daily dose can easily be adjusted at home without a new prescription being issued if the dose changed. The daily dose was therefore often not recorded. In about 85% of the prescriptions of VKAs the daily dose was missing. The few daily doses recorded were not assumed to be consistent over the prescription length and were not used to calculate prescription lengths as done for available records for DOACs. For VKAs and NSAIDs, missing prescription lengths were replaced by the samples median prescription length, which was considered most representative of an average user. The median length was generated from time between prescription dates if missingness for daily dose records was ≥50%, as was the case for VKA prescriptions. If missingness of daily doses was <50%, the median of available calculated lengths was used, as it was the case for NSAIDs. Because missing daily doses for DOACs were already replaced by the standard BNF dose, this step was not required for DOACs.

For each prescription, the stop date was generated by adding the prescription length to the date the prescription was issued. Continuous prescription periods started with the issue of the first prescription and ended with the calculated stop date of the last prescription plus the 30‐day grace period. The described approach to identify stop dates and then periods of continuous prescriptions was considered reasonable by pharmacists and epidemiologists involved.

## Appendix E ICD‐10 code lists for outcomes

This section contains ICD‐10 codes used to identify outcomes. Read codes used to identify patients on treatment with oral anticoagulants and NSAIDs are not reported here but available from the authors on request. Read codes for baseline characteristics are also available on request.

**TABLE E1:** ICD‐10 codes used to identify outcomes in Hospital Episodes Statistics (HES)

ICD‐10	Description	Type
I61	Intracerebral haemorrhage	Stroke, MB
I61.1	Intracerebral haemorrhage in hemisphere, cortical	Stroke, MB
I61.2	Intracerebral haemorrhage in hemisphere, unspecified	Stroke, MB
I61.3	Intracerebral haemorrhage in brain stem	Stroke, MB
I61.4	Intracerebral haemorrhage in cerebellum	Stroke, MB
I61.5	Intracerebral haemorrhage, intraventricular	Stroke, MB
I61.6	Intracerebral haemorrhage, multiple localized	Stroke, MB
I61.8	Other intracerebral haemorrhage	Stroke, MB
I61.9	Intracerebral haemorrhage, unspecified	Stroke, MB
I63	Cerebral infarction	Stroke
I63.0	Cerebral infarction due to thrombosis of precerebral arteries	Stroke
I63.1	Cerebral infarction due to embolism of precerebral arteries	Stroke
I63.2	Cerebral infarction due to unspecified occlusion or stenosis of precerebral arteries	Stroke
I63.3	Cerebral infarction due to thrombosis of cerebral arteries	Stroke
I63.4	Cerebral infarction due to embolism of cerebral arteries	Stroke
I63.5	Cerebral infarction due to unspecified occlusion or stenosis of cerebral arteries	Stroke
I63.8	Other cerebral infarction	Stroke
I63.9	Cerebral infarction, unspecified	Stroke
I64	Stroke, not specified as haemorrhage or infarction	Stroke
G45	Transient cerebral ischaemic attacks and related syndromes	Stroke
G45.0	Vertebro‐basilar artery syndrome	Stroke
G45.1	Carotif artery syndrome (hemispheric)	Stroke
G45.2	Multiple and bilateral precerebral artery syndromes	Stroke
G45.3	Amaurosis fugax	Stroke
G45.8	Other transient cerebral ischaemic attacks and related syndromes	Stroke
G45.9	Transient cerebral ischaemic attack, unspecified	Stroke
G46	Vascular syndromes of brain in cerebrovascular diseases	Stroke
G46.0	Middle cerebral artery syndrome	Stroke
G46.1	Anterior cerebral artery syndrome	Stroke
G46.2	Posterior cerebral artery syndrome	Stroke
G46.3	Brain stem stroke syndrome	Stroke
G46.4	Cerebellar stroke syndrome	Stroke
G46.5	Pure motor lacunar syndrome	Stroke
G46.6	Pure sensory lacunar syndrome	Stroke
G46.7	Other lacunar syndromes	Stroke
G46.8	Other vascular syndromes of brain in cerebrovascular disease	Stroke
I26.0	Pulmonary embolism with acute cor pulmonale	SE
I26.9	Pulmonary embolism without acute cor pulmonale	SE
I74.0	Embolism and thrombosis of abdominal aorta	SE
I74.1	Embolism and thrombosis of other and unspecified parts of aorta	SE
I74.2	I74.2 embolism and thrombosis of arteries of the upper extremities	SE
I74.3	I74.3 embolism and thrombosis of arteries of the lower extremities	SE
I74.4	I74.4 embolism and thrombosis of arteries of extremities, unspecified	SE
I74.5	I74.5 embolism and thrombosis of iliac artery	SE
I74.8	I74.8 embolism and thrombosis of other arteries	SE
I74.9	I74.9 embolism and thrombosis of unspecified artery	SE
I85.0	Oesophageal varices with bleeding	GIB, MB
K22.8	Haemorrhage of oesophagus	GIB, MB
K25.0	Gastric ulcer, acute with haemorrhage	GIB, MB
K25.1	Gastric ulcer, acute with perforation	GIB, MB
K25.2	Gastric ulcer, acute with both haemorrhage and perforation	GIB, MB
K25.4	Gastric ulcer, chronic or unspecified with haemorrhage	GIB, MB
K25.5	Duodenal ulcer, chronic or unspecified with perforation	GIB, MB
K25.6	Chronic or unspecified with both haemorrhage and perforation	GIB, MB
K26.0	Duodenal ulcer, acute with haemorrhage	GIB, MB
K26.1	Duodenal ulcer, acute with perforation	GIB, MB
K26.2	Duodenal ulcer, acute with both haemorrhage and perforation	GIB, MB
K26.4	Duodenal ulcer, chronic or unspecified with haemorrhage	GIB, MB
K26.5	Duodenal ulcer, chronic or unspecified with perforation	GIB, MB
K26.6	Chronic or unspecified with both haemorrhage and perforation	GIB, MB
K27.0	Peptic ulcer, acute with haemorrhage	GIB, MB
K27.1	Peptic ulcer, site unspecified, acute with perforation	GIB, MB
K27.2	Peptic ulcer, acute with both haemorrhage and perforation	GIB, MB
K27.4	Peptic ulcer, chronic or unspecified with haemorrhage	GIB, MB
K27.5	Peptic ulcer, site unspecified, chronic or unspecified with perforation	GIB, MB
K27.6	Chronic or unspecified duodenal ulcer with both haemorrhage and perforation	GIB, MB
K28.0	Gastrojejunal ulcer, acute with haemorrhage	GIB, MB
K28.1	Gastrojejunal ulcer, acute with perforation	GIB, MB
K28.2	Acute gastrojejunal ulcer with both haemorrhage and perforation	GIB, MB
K28.4	Gastrojejunal ulcer, chronic or unspecified with haemorrhage	GIB, MB
K28.5	Gastrojejunal ulcer, chronic or unspecified with perforation	GIB, MB
K28.6	Chronic or unspecified ulcer with both haemorrhage and perforation	GIB, MB
K29.0	Acute haemorrhagic gastritis	GIB, MB
K66.1	Haemoperitoneum	GIB, MB
K92.0	Haematemesis	GIB, MB
K92.1	Melaena	GIB, MB
K92.2	Gastrointestinal bleed, unspecified	GIB, MB
J94.2	Haemopneumothorax	MB
H31.3	Choroidal haemorrhage and rupture	MB
H43.1	Vitreous haemorrhage	MB
H45.0	Vitreous haemorrhage in diseases classified elsewhere	MB
R04	Haemorrhage from respiratory passages (epistaxis, throat, cough with haemorraghe)	MB
R04.1	Haemorrhage from throat	MB
R04.2	Haemoptysis	MB
R04.8	Haemorrhage from other sites in respiratory passages	MB
R04.9	Haemorrhage from respiratory passages, unspecified	MB
R31	Unspecified haematuria	MB
R58	Haemorrhage, not elsewhere classified	MB
M25.0	Haemarthrosis (bleeding into joint spaces)	MB
N02	Recurrent and persistent haematuria	MB
K62.5	Haemorrhage of anus and rectum	MB
K55.21	Angiodysplasia of colon with bleed	MB

GIB: gastrointestinal bleeds including ulcer perforations referred to as serious GI events; MB: major bleeding events; SE: systemic embolism.

## Appendix F Sensitivity analysis

**TABLE F1:** Cox regression model results of sensitivity analyses for each outcome with time‐dependent NSAID exposure

Outcome (*n =* number of patients in cohort)	Number of outcomes		Person time		HR (95% CI)^a^
	Exposed to NSAIDs	Unexposed to NSAIDs	Exposed to NSAIDs	Unexposed to NSAIDs	Exposed vs. unexposed
**GI bleeding**					
Base case (*n =* 6354)^b^	13	53	635	7710	3.01 (1.63; 5.55)
*Assumptions on data preparation*					
60‐d grace period (*n =* 9824)	26	133	1509	18 281	2.60 (1.70; 3.97)
NSAID washout assessment window 6 mo (*n =* 5308)	13	41	496	6880	4.86 (2.58; 9.15)
NSAID washout assessment window 1 mo (*n =* 11 460)	24	85	1190	11 784	2.54 (1.60; 4.02)
Excluding patients with GI bleeding (*n =* 6180)	13	40	620	7544	4.04 (2.14; 7.61)
*Assumptions around confounding variables*					
Stabilised IPTW instead of PSM (*n =* 109 894)	16	1067	626	103 975	2.47 (1.40; 4.35)
GPA not used as variable in PSM (*n =* 6354)	13	56	635	7674	2.96 (1.61; 5.44)
Cox regression with time‐varying confounder (*n =* 6354)^c^	13	53	635	7710	3.10 (1.67; 5.75)
**Stroke**					
Base case (*n =* 6342)^b^	13	62	634	7779	2.71 (1.48; 4.96)
*Assumptions on data preparation*					
60‐d grace period (*n =* 9796)	21	159	1509	18 338	1.80 (1.14; 2.84)
NSAID washout assessment window 6 mo (*n =* 5298)	11	58	495	6894	2.69 (1.41; 5.16)
NSAID washout assessment window 1 mo (*n =* 11 448)	18	95	1192	11 819	1.93 (1.16; 3.22)
Excluding patients with stroke (*n =* 5926)	8	59	586	7015	1.67 (0.79; 3.50)
*Assumptions around confounding variables*					
Stabilised IPTW instead of PSM (*n =* 109 894)	16	1570	627	103 779	1.72 (0.95; 3.09)
GPA not used as variable in PSM (*n =* 6342)	13	60	634	7631	2.76 (1.51; 5.06)
Cox regression with time‐varying confounder (*n =* 6342)^d^	13	62	634	7779	3.21 (1.53; 6.70)
**Major bleeding**					
Base case (*n =* 6288)^b^	28	130	621	7532	2.77 (1.84; 4.19)
*Assumptions on data preparation*					
60‐d grace period (*n =* 9654)	55	326	1465	17 713	2.14 (1.60; 2.85)
NSAID washout assessment window 6 mo (*n =* 5246)	24	115	486	6593	2.79 (1.79; 4.35)
NSAID washout assessment window 1 mo (*n =* 11 390)	54	228	1168	11 385	2.19 (1.62; 2.95)
Excluding patients with major bleeding (*n =* 5818)	23	116	571	7056	2.47 (1.57; 3.89)
*Assumptions around confounding variables*					
Stabilised IPTW instead of PSM (*n =* 109 894)	31	2798	622	102 735	1.87 (1.26; 2.78)
GPA not used as variable in PSM (*n =* 6288)	28	127	621	7505	2.75 (1.82; 4.16)
Cox regression with time‐varying confounder (*n =* 6288)^c^	28	130	621	7532	2.77 (1.83; 4.21)
**Systemic embolism**					
Base case (*n =* 6350)^b^	<5^e^	10	636	7715	3.02 (0.82; 11.07)
*Assumptions on data preparation*					
60‐d grace period (*n =* 9838)	7	41	1518	18 764	1.99 (0.89; 4.48)
NSAID washout assessment window 6 mo (*n =* 5304)	<5^e^	12	497	6861	1.92 (0.43; 8.62)
NSAID washout assessment window 1 mo (*n =* 11 456)	8	34	1192	11 734	1.60 (0.73; 3.50)
Excluding patients with systemic embolism (*n =* 5820)	<5^e^	8	585	7198	2.75 (0.57; 13.16)
*Assumptions around confounding variables*					
Stabilised IPTW instead of PSM (*n =* 109 894)	<5^e^	313	628	104 408	1.93 (0.59; 6.32)
GPA not used as variable in PSM (*n =* 6350)	<5^e^	13	636	7664	2.40 (0.68; 8.51)
Cox regression with time‐varying confounder (*n =* 6350)^d^	<5^e^	10	636	7715	2.97 (0.81; 10.95)

^a^ Unadjusted for all analyses but the Cox regression with time varying confounders; ^b^ base case: 30‐day grace period, 3 months NSAID washout period, PSM not including GPAs and using missing records as a separate missing category for each variable; ^c^ HR adjusted for: age ≥65 years, renal disease, chronic liver disease, uncontrolled blood pressure, bleeding, peptic ulcer, anaemia, alcoholism, aspirin, antiplatelet, antidepressants and corticosteroids; ^d^ HR adjusted for: age≥75 heart failure, stroke, hypertension, perivascular disease, coronary heart disease and diabetes; ^e^ cell counts <5 are masked to avoid that patients can be identified from this analysis; GPA: gastroprotective agent; HR: hazard ratio; IPTW: inverse probability of treatment weighting; NSAID: nonsteroidal anti‐inflammatory drug; PSM: propensity score matching.

## Appendix G E‐values

**TABLE G1:** E‐values compared with the impact of risk factors for bleeding outcomes conditional on NSAID exposure from Cox proportional hazard model in the base case analysis

Parameter	GI bleeding	Major bleeding
E‐value (mean, lower bound)	5.45, 2.64	4.84, 3.08
*Risk factors, HR (95% CI)* ^*a*^		
Renal disease	1.12 (0.27; 4.56)	1.18 (0.48; 2.87)
Chronic liver disease	N/A^b^	N/A^b^
Age ≥65 y	1.60 (0.79; 3.27)	1.37 (0.88; 2.15)
Uncontrolled blood pressure (>160 mmHg)	1.49 (0.54; 4.11)	1.03 (0.48; 2.21)
Alcohol dependence	2.14 (0.67; 6.83)	1.73 (0.76; 3.91)
Bleeding	1.24 (0.65; 2.38)	1.62 (1.10; 2.39)
Peptic ulcer	2.80 (1.28; 6.13)	1.97 (1.09; 3.54)
Anaemia	1.08 (0.52; 2.27)	1.25 (0.79; 1.98)
Aspirin	1.21 (0.69; 2.1)	1.12 (0.78; 1.63)
Antiplatelet	2.15 (0.92; 4.99)	1.12 (0.54; 2.27)
Antidepressants (SSRI, TCA)	1.90 (1.11; 3.23)	1.58 (1.10; 2.27)
Corticosteroids	0.79 (0.32; 1.98)	0.90 (0.51; 1.59)

^a^ Variables available in the dataset and identified as time‐dependent confounders in sensitivity analysis;

^b^ counts too low and omitted; CI: confidence interval; HR: hazard ratio; NSAID: nonsteroidal anti‐inflammatory drug; SSRI: selective serotonin reuptake inhibitor; TCA: tricyclic antidepressant.

**TABLE G2:** E‐values compared with the impact of risk factors for thromboembolic outcomes^a^ conditional on NSAID exposure from Cox proportional hazard model in the base case analysis

Parameter	Stroke
E‐value (mean, lower bound)	4.84, 2.32
*Risk factors, HR (95% CI)* ^*b*^	
Heart failure	1.13 (0.62; 2.05)
Stroke/transient ischaemic attack	3.81 (2.42; 5.99)
Age≥75	1.06 (1.03; 1.09)
Hypertension	2.77 (1.60; 4.82)
Peripheral artery disease	2.24 (0.91; 5.56)
Coronary heart disease	2.46 (1.57; 3.85)
Diabetes	1.17 (0.68; 1.04)
Female	1.53 (0.98; 2.39)

^a^ Systemic embolism not considered because the base case HR was not significant;

^b^ variables of the CHA2DS2‐VASc available in the dataset; CI: confidence interval; HR: hazard ratio; NSAID: nonsteroidal anti‐inflammatory drug.
